# Willingness of postmenopausal women to participate in a study involving local vaginal oestrogen treatment as an adjunct to pelvic organ prolapse surgery: a qualitative study

**DOI:** 10.1007/s00192-020-04480-5

**Published:** 2020-08-25

**Authors:** Tina Sara Verghese, Abigail Merriel, Lisa Leighton, Pallavi Latthe

**Affiliations:** 1 Clinical Research Fellow Institute of Metabolism and System Research, College of Medical & Dental Sciences, University of Birmingham, Birmingham, UK; 2 Population Health Sciences, University of Bristol, Bristol, UK; 3 Birmingham Clinical Trials Unit, Birmingham, UK; 4 Birmingham Women’s and Children’s NHS Foundation Trust, Birmingham, UK

**Keywords:** Postmenopausal women, Oestrogen, Qualitative design

## Abstract

**Background:**

Pelvic organ prolapse (POP) negatively affects many women’s quality of life. The ability to develop improved therapeutic approaches for POP patients is hampered by low patient recruitment and retention rates in clinical trials.

**Objective:**

Our objective was to explore the motivational factors and barriers to recruitment and participation in clinical trials among postmenopausal women with POP who are intending to have surgical management.

**Design:**

Qualitative study based on in-depth face-to-face interviews with postmenopausal women attending urogynaecology clinics in the UK intending to have surgical management for pelvic organ prolapse. These women were eligible to participate in the on-going clinical trial on the use of local vaginal oestrogen as an adjunct to surgical treatment. Twenty-two postmenopausal women aged 52–76 years were interviewed. Interviews were analysed using thematic analysis method.

**Results:**

Many women participated because of altruistic motivations; however, we found that clarity of information provided, timing of approach and acceptability of study design played a pivotal role in women. Of the women who declined participation, the following themes emerged: uncertainty of the investigational product, fear of experimentation, logistical concerns and regret that their condition was trivialised at an early stage.

**Conclusion:**

We have gained a valuable insight into women’s views and experience in the decision making process. Understanding the elements that will enhance trial participation such as clarity of information provided, balance between professional guidance whilst maintaining equipoise, easy access to trial teams and timing of approach will ultimately enable us to improve our recruitment to clinical trials.

## Introduction

Pelvic organ prolapse is a chronic condition affecting millions of women. The condition negatively impacts on their quality of life through effects on their urinary and bowel symptoms, body image and chronic backache [1]. The severity of symptoms is not, however, correlated with the level of POP. Women are offered varied treatment options from expectant, conservative and surgical management.

One of the main recommendations of the UK government Life Science Strategy was for the National Health services (NHS) to respond to the growing readiness of patients to participate in research studies [2]. The strategy recommended researchers to respond to patient choices to enhance participation within a trial. However, there is extensive evidence from the NHS acute hospital sector that poor patient recruitment or retention of patients to clinical research is widespread, leading to delays in commencement or completion of both academic and commercially funded research [3]. This results in wastage of public resources and opportunities for patient participation.

We performed a study to assess the feasibility of patient screening and recruitment process to study the effectiveness of Local Oestrogen Treatment in Postmenopausal Women Undergoing Pelvic Organ Prolapse Surgery (LOTUS) 10.1186/ISRCTN46661996. This study was in preparation for a large definitive randomised controlled study to determine whether pre- and postoperative local oestrogen treatment is more effective in improving prolapse-related patient-reported outcomes and reducing recurrence of prolapse symptoms when compared to no treatment.

The research team identified the women eligible to participate in the feasibility trial by screening the GP referral letters. Potential eligible women were sent patient information leaflets before the clinic appointments in order to give the women an opportunity to consider participation in the clinical trial. The trial team comprised of a research fellow, research nurses and urogynaecologist from district and tertiary hospitals across the UK. Once eligibility was reconfirmed, women were given sufficient time to obtain informed written consent and collect baseline data. Randomisation was performed using a web-based central randomisation system (via Birmingham Clinical Trials Unit) to allocate patients to either oestrogen or no treatment in a 1:1 ratio. Minimisation was used to achieve balance between age (< 65 years or ≥ 65 years), parity (≤ 2 or > 2 vaginal births) and maximum stage of prolapse (I, II or III/IV).

Women allocated to oestrogen (oestradiol hemihydrate 10 mcg vaginal pessaries; Vagifem™, Novo Nordisk) were instructed to use the oestrogen pessaries 6 weeks prior to surgery (once daily for 2 weeks and twice weekly for 4 weeks) up to the night before surgery. Treatment was restarted 6 weeks postoperatively, administering twice weekly for 20 weeks. Women were encouraged to insert the pessaries into the vagina at the same time of day. However, if a dose was missed, patients were advised it should be administered as soon as possible thereafter, provided the next dose was not due. Participants allocated no treatment received the usual care of the randomising centre. The surgical approach to POP repair was at the discretion of the urogynaecological surgeon. The researchers conducting the qualitative interviews were blinded to the participant’s intervention allocation within the study.

Approximately quarter of women opt for surgical repair of POP [4]. There are few studies seeking to explore the experience of postmenopausal women with POP planning on undergoing surgical repair. Therefore, alongside the LOTUS feasibility study, we undertook a qualitative study with the objective of exploring the factors that motivate this group of women to participate in clinical trials. Some women see POP as a sensitive and embarrassing condition; therefore, to prepare for our planned future trial, we aimed to identify barriers to recruitment and participation in clinical trials among postmenopausal women with pelvic organ prolapse intending to have surgical management.

## Methods

We conducted semi-structured, in-depth, audio-recorded interviews with postmenopausal women eligible to participate in the LOTUS study (Table 1). Interviews were conducted from October 2015 until July 2016 from Birmingham Women’s and Children’s NHS Foundation Trust. The research team proposed a priori sample size at the time of initial proposal of ten interviews with a further stopping criterion of five interviews. The essence of these interviews was to obtain information and shared beliefs until data saturation was achieved [5–8]. The research team ultimately interviewed 22 women individually. Along with this a focused group interview (10 women) was used to expand on and verify the emerging themes from individual themes.

**Table 1:** Qualitative Interview guide

Regarding pelvic organ prolapse	**When did you first start experiencing symptoms of prolapse?** *• How did it affect your day-to-day life?* *•* **What did you understand about prolapse** *• When were you diagnosed?* *• How did you feel about the diagnosis?* *• Did you try another treatment?* **Tell me about your experiences of using pessaries/ physiotherapy for prolapse** *• How effective was this treatment?*
Views on Medical Trials [General]	**What do you think about medical trial?** *• for individuals?* *• for medical science?* **What do you think about randomisation?** *• Understandings of randomisation/how treatment is allocated* *• Is randomisation acceptable to you?* *• Is the possibility of not getting treatment acceptable?*
Views on participation in LOTUS study	**What are your thoughts about the trial?** *• NB: Expand into an open-ended discussion about the trial* *° Hopes for the trial* *° Concerns about the trial* **What would motivate/motivated you to take part in the trial?** *What would you hope to get out of participating in this study?* **What are your main concerns about participating in the trial?** *•* ***What would be a barrier to you participating?*** *° Personal factors?* *• Past experiences with treatments or trials* *• Time or travel costs* *° Trial factors?* *• Concerns about treatment availability* *• Concerns about treatment choice and randomisation* **What did you think about the study after reading the patient information leaflet for the study?** *° Did you feel the leaflet gave you enough information?*
Experiences of hormone replacement treatment (HRT)	**What do you understand by HRT:** *• Have you used HRT before? For how long?* *• Do you have any concerns regarding HRT?* *• Have used oestrogen pessaries or creams in the past? Which would you prefer to use?*

Of the 22 women interviewed, 7 who had initially declined participation in the LOTUS trial were willing to take part in the qualitative arm of the study. Participation in the qualitative arm of the study was purely voluntary among those who participated or declined participation in the LOTUS trial. The study aimed for a diverse, maximum variation sample of participants. Participants were sent a patient information sheet, had the study explained to them and signed a consent form before the interview. The women who consented to participate in the qualitative arm of the study were given the choice of place where they would prefer to have the interview in order to make sure that the women were most comfortable and free to voice their thoughts. Women were interviewed in informal non-clinical settings such as quiet rooms in hospitals or at the comfort of their own homes via teleconference sessions. This allowed the women to chose the most convenient time and place; therefore, they never felt pressured or rushed. The interview had two parts:

- An unstructured narrative section, in which participants were asked to tell their own story with as little interruption as possible, to capture their own accounts of their experience with POP, what brought them to the hospital and aspects of the trial that they felt were important to them.
- A series of prompts, used by the interviewer to explore particular issues further in a semi-structured part of the interview

The interviews lasted 45 to 90 min each. The interviews were transcribed verbatim and analysed thematically using the organisational support of NVivo 10 software. Transcripts were read and re-read carefully by the interviewer, and a coding framework was developed. A second researcher checked the transcripts (LL) and independently coded the first few interviews; results were compared and discussed. The coding framework was revised and further developed. A qualitative interpretative approach was utilised, combining thematic analysis with constant comparison continuously looking for anticipated and emergent themes [6, 7]. Field notes were made after every interview process in order for the interviewer to capture the understanding and body language aspects of patient during the interview when talking on certain aspects of the condition and the trial. A modified grounded theory approach using the “one sheet of paper” method ensured that all the coded extracts within the theme were included and compared in the analysis. The approach ensured that every instance and nuance was considered importantly including deviant cases. Qualitative data collection and analysis often proceed iteratively to achieve data saturation, with analytic categories saturated when data from new interviews do not add any more to the analysis [6, 7].

## Results

Twenty-two women with symptomatic POP were recruited for the qualitative study. Symptomatic POP was defined as presence of a vaginal bulge and/or other symptoms from the bladder or bowel. The women were assessed using the POP-Q classification system [9]. Recruitment was according to purposive sampling, i.e., the greatest variation of characteristics such as age, parity, BMI and stage of prolapse in order to capture wide narratives from the informants (Table 2). The results are summarised in Table 3.

**Table 2:** Background characteristics of participants

Characteristics of participants	Total	*n* = 22 (%)
Age	< 65 years	15(68%)
	≥ 65 years	7(14%)
Ethnic group	White	18 (81%)
	Asian	3 (13%)
	Black	1 (4%)
	Mixed	–
Parity	≤ 2	12(54%)
	> 2	10(50%)
BMI (mg/kg^2^)	Mean (SD)	28.1
Maximum stage of prolapse	Stage I	3(14%)
	Stage II	9(41%)
	Stage III/IV	10(45%)
Marital status	Married	15(68%)
	Single	3(14%)
	Widow	4(18%)
Occupation	Employed	15(68%)
	Retired	7(32%)

**Table 3:** Summary of results

Facilitating factors to recruitment	Barriers to recruitment
Altruism	Uncertainty of treatment proposed
Simplicity of trial protocol	Logistical factors—number of clinic appointments, parking charges,transport dependence, leave from work
Clear and succinct patient information leaflets	False perception of delay in receiving treatment if participates in clinical trial
Easy accessibility to research teams via contact telephone numbers and email addresses	Time constraints for patients
Active clinician input and collaboration with trial units	Trivialisation of condition by primary care
Screening GP referral forms prior to clinic appointments	
Sending information leaflets to potential participants before the clinic appointment	

### Factors that facilitated recruitment

#### Theme 1: probably help another woman

The majority of women who were approached to participate in the LOTUS trial were willing, as they wanted to help other women and hoped that their contribution and/or participation would help other women in their similar situation. Interestingly, it was the group of women who had struggled for many years with prolapse who readily wanted to participate rather than those who received a new diagnosis. The women who declined regretted that they did not request to be seen at tertiary care earlier and felt that primary care did not offer them much help with their symptoms.

*P3: As I sat in the waiting room just looking at the trial poster and after reading through the leaflet, there was this inclining in me to participate, I have had just enough with my symptoms, if I can participate in this trial and if it would help another woman I would be really happy.*

*P5: I’m retired now and I have participated in other trials and I think this study really makes sense, I think this will make a change for the future generation.*

*P6: I am 76 years old and suffered with a prolapse for so many years, I know ageing is a process and having a prolapse may be part of it, but I think more women should talk about it…These are present day problems and studies like this will start conversations, which I think is essential. You do not often hear women’s hour talking about vaginal prolapse (laughs). Yes, I would definitely take part, I hope the study triggers women to talk more about vaginal prolapse, this should help.*

*P11: I wish I did not carry on with these pelvic exercises, now the gynaecologist offers me surgery. There should be some time limits in place. My doctor examined me once and no further, perhaps I could have avoided surgery! I rather just get on have surgery with no further delays in my treatment.*

#### Theme 2: Easy to understand protocol and research team accessibility

This study reinforces that clear and easy to understand protocols played a vital role in encouraging women to participate in the study. Reading and understanding a study consent form has been shown to be critical to enrolment in a trial. The women within the study felt that they had the ability to access the trial team at any point during the study and felt reassured. This was a safety net that many of them felt convinced would help them through the process without just becoming a trial number to the research. Many felt that interaction between themselves and the trial team gave them the confidence to complete the trial from start to end.

*P12: I received the leaflet prior to me coming for my appointment; it was quite straightforward and simple. I had a few questions, which the researcher answered for me.*

*P10: The clinician made it very easy for me to understand the process; we had a really long chat. I was able to make my mind very easily. I trust the team here, no matter how many times I asked questions the team were very approachable. The clinic appointment took nearly 45 min but they put the effort in making sure that I understood what I was getting myself into.*

*P4: I contacted the research team and spoke to the trial team when I went home, I felt this was a brilliant service. When you sit in a clinic environment you definitely feel on the spot and though there were a number a questions going through my mind, in clinic I did not feel I could make a decision. But when I went home spoke to my husband and then contacted the trial team I felt more confident.*

*P6: I think this trial did not look so complicated. Yes, it is an open trial and that’s fine in my eyes…at least I know what I am going to be taking. The researchers were thorough and they were easily contactable. The were able to listen to my concerns and really took a few of my suggestions which made feel like my voice was valued and I wasn’t another trial number.*

With this study being a feasibility study, many of the participants were free to voice their views on the protocol, design and access to research team. These women felt part of the research team that shaped the study from a patient’s point of view. This even led two of participants becoming part of the patient and public involvement (PPI) group.

#### Theme 3: Senior clinician input and collaboration between the research and clinical teams

The recruitment process itself is often complex and involves several linked activities performed by clinical and research staff within and between different centres. We found that the women who participated in the trial felt that input from the clinicians gave them a security and confidence in enrolling in a randomised control study.

*P20: I found that talking to the clinician made be more confident in the trial. She seemed to explain the ins and outs of this trial. She helped understand what they were looking for and why they were conducting the clinical trial. I trust the clinical team; I mean they were the ones that took my symptoms seriously and I am finally being offered some kind of surgery.*

*P15: The clinician really understood my condition; she explained what was happening and the reason why I am probably having these symptoms. I guess this oestrogen might help; that’s if I get that and if not then I am only being offered what the rest the women with my condition are receiving on the NHS.*

### Two-step approach

The researchers in this study screened the GP referral letters and were able to identify potential women who could be invited to participate in the study. The potential women were sent patient information leaflets prior to them attending their clinic appointments. This process assisted in preparing women regarding the clinical discussion and the clinical trial.

*P2: I received a leaflet along with my clinic letter; I was able to read through this prior to me attending this appointment. This leaflet was quite helpful and I was able to identify many of my symptoms from just reading the leaflet. I felt more confident; I knew I was attending the right clinic. I did read about the trial as well; I was able to chat with my husband about the study and ask a few more questions to the clinician regarding it.*

*P7: I think this method of sending the details about the study is helpful; it really saves time. I had time to think about the study and I was able to make up my mind regarding participation.*

*P14: I had loads of questions before I met the doctor, I even wrote my queries on the leaflet. You need the time to think about these things.*

*P17: I knew I was in the right place, I took my time deciding whether I should participate. I read the information and spoke to the clinician. I went home and then after discussing with my family, I was able to contact the trial team and informed them that I wanted to participate. I think it’s easier to make up your mind when you are in your own home. Loads of things went through my head when I was in clinic. We talked about so many things, the surgery and the study.*

## Barriers to recruitment

### Theme 4: Uncertainty

The major barrier to this study was the uncertainty of the study product. The women who declined participation expressed that they had a fear of experimentation and were concerned of developing cancer. The number of discrepancies regarding hormone replacement therapy fuelled this uncertainty. Participants felt that their GPs were not confident in prescribing long-term hormone replacement therapy. Few participants did not like to be “a guniea pig” in the process of the trial. The conflicting information that they have received in the past with regard to HRT made them worry about participation.

*P14: I am not sure about hormone replacement; I had spoken with my GP on a previous occasion regarding taking oestrogen replacement and he did not want me to have therapy for long term.*

*P10: I worry about developing cancer; I know this is a low dose but who knows? Why would you want to put yourself in such a position?*

*P3: This is conflicting information; you hear about the ill effects of HRT and its relation with cancers like breast. I am here for is being described as a chronic problem; if the surgery can fix my prolapse why would I take any other medication? I would be quite anxious of developing any side effects.*

### Theme 5:Logistic factors

There were various logistical factors that were highlighted by the women who felt that inhibited them for participating in the trial.

*P20: Coming to clinic appointments are really very difficult for me; I need my daughter to be available to bring me to the clinic appointments. I do not want to be more of a burden to her. I guess the lesser appointments the better.*

*P16: The parking charges in this hospital are really ridiculous; I mean if I participate it would mean that I would probably be here longer and if participation would wave these charges perhaps I would consider participating.*

*P14: So many clinic appointments; I think the lesser time I am at the hospital the better! I think I am always in the hospital and lesser at home (laughs).*

*P17: I need to be there for my husband; he needs round the clock care. I would not have come to this clinic; it has only been because I started to have some bleeding that I did come to this appointment. I would have just continued to ignore my symptoms otherwise. I do not think I can possibly participate at this point in my life; there is just too much going on.*

### Theme 6: Time between intervention and surgery

As per the study protocol, after randomisation participants in the intervention arm were requested to commence medication for a period of 6 weeks prior to their surgery. Some of the women who were approached felt that they did not want to wait for approximately 6–8 weeks for surgery. These women perceived that time between commencing the investigational product and surgery was a delaying factor and thought this would disadvantage them on the waiting list for surgery.

*P6: I was told that I require surgery; I do not want to wait for another 2 months until I have surgery.*

*P15: I have put with these symptoms far too long, I would just like to get on and have the surgery now. I think I have postponed my treatment far too long; I think if I do participate I will just be delaying the process.*

### Theme 7: Trivialisation of the condition

During the interview process, many women voiced their thoughts regarding their condition and symptoms. De-prioritisation of their own symptoms for several years before they presented to medical team was recognised as a theme among the women. Some felt embarrassed to come forward while others felt regret for leaving their symptoms for so long. Interestingly, some women were not self-aware of the worsening of their condition. They were not sure what impact prolapse could have on their bladder or bowel. The recognition of these worsening symptoms would assist the women and their GPs for a referral to tertiary care.

*P4: I went to my GP years ago and at that point she did not say that my prolapse would worsen; all she said was to do pelvic floor exercise. I think doctors have been telling me to do this since I had my son nearly 30 years ago. I did not realise I would need a surgery.*

*P8: I always had symptoms of prolapse and in fact I was so embarrassed; I avoided going to the gym I was so worried I would leak or others could notice my prolapse. Everyone said it was part of ageing process…maybe I should have gone to the doctor earlier.*

*P10: I am so irritated I always kept voicing my concern with my GP. I do not think any one examined me in the past. Now I am being told that I would require surgery. I just want to get the surgery over with. I wish some had paid more attention to my symptoms earlier.*

The initial and emerging codes have been complied in Table 4.

**Table 4:** Coding index

Themes	Initial categories
Clarity in information	Trial teams gave maximum information Individualised their concerns Easy to understand Not very complex patient information leaflet Faith in clinical teams Co-ordination between clinical and research teams
Timing of approach and environment	Two step approach Given enough time Ability to contact trial teams Easier to make a decision at home Dedicated clinics for the trial purposes Opportunities to ask questions
Acceptability of study design	Understand the process of randomisation Recall the design Understand and maintain equipoise
Uncertainty	Fear of cancer Varied information online Unpredictable outcomes Concerns of side effects
Logistical concerns	Dependent on family and friends No monetary incentives Timing between intervention and surgery Other co-morbidities Multiple appointments
Trivialisation	Let down by clinical team Prioritising other family issues over symptoms Carer for family members Embarrassed by the condition/ symptoms Ageing process

## Discussion

We have sought to produce an understanding of 22 individuals with POP and their experience within the trial and their decisions making process as to what compelled them to take part or refuse participation in the trial. We have demonstrated that there was considerable variation among our participants, but there were some strong common themes as well without downplaying the uniqueness of each person’s view.

This study has helped us to understand factors that would be likely to motivate or detract from patient participation and retention within our planned trial. The factors that we identified were part of procedural, communication and resource issues. The minimum contact must have a very specific structure and the research team must follow the structured guidelines until the woman feels sufficiently comfortable. There must be a minimum adherence plan for the entire multicentre clinical trial in the developmental stages of the trial [10].

During the trial, researchers should be perceptive to the feedback and information received from patients at the time of recruitment. Despite numerous strategies present in the literature, gaps remain. Gul and Ali [11] identified that the majority of recommended interventions for enhancing the recruitment and in clinical studies are ‘piecemeal’ and take little account of how local practices of recruitment work influence the effectiveness of such interventions [11].

Taking part in research is a complex decision. Multiple factors play a role in decision-making. Altruism may have existed in many participants and is seen across all clinical studies [12]; however, in this group there was also an element of their symptoms finally receiving recognition and acknowledgement of a problem, which was ultimately taken seriously when they enrolled into the trial. The majority felt that their symptoms had been trivialised in the past. They voiced the need for forums for women to talk openly about POP and welcomed research in this area. Prolapse symptoms hindered their quality of life and with an ageing population, there are necessities to not only prolong life expectancy but equally importance to quality of life. Participants experienced barriers to trial participation including additional demands such as attending appointments and associated time, effort or financial costs, discomfort associated with trial procedures, the risk of not being allocated to their preferred treatment and uncertain outcomes. Likewise, Fogel et al. reported burdens including potential side effects from treatment, additional tests that would have to be endured, financial concerns (including loss of job support and work disruption) and a general worry about the unknown future, including whether or not the study drug assigned would be beneficial [13, 14]. Sometimes patients are not presented with a clear rationale for why their participation is important and receive minimal feedback.

Women who received the leaflets prior to coming to their designated clinic appointment found it easier to make their decision on trial participation. This two-step approach of screening potential participants and sending out information to the women prior to as well as having dedicated clinics for potential participants gave the optimum time for women to think over their options [15]. Screening patient records, identifying eligible patients, preparing recruitment material and ensuring that the relevant clinicians were informed about the study, were useful strategies practised by the research teams. Similar strategies are echoed in various other clinical trials as well [16, 17]. Chhatre et al. found that contacting potential participant prior to clinical appointment helped streamline the recruitment process [18]. Furthermore, an easily accessible research team and the quality of information provided to these patients gave the women an added confidence not only with the clinician but with the research team as well.

The practicalities and co-ordination of balanced information provision for patients about both treatments can be challenging. Clinicians may be comfortable explaining interventions they routinely deliver but they may well be less confident conveying the effectiveness of treatments outside their specialist remit. However, the research team were able to maintain equipoise better than the clinicians in informing patients of the treatment choices. The research team at several points had anxiety regarding the eligibility of women into the study.

In the LOTUS trial there were a few patients who were flagged at multi-disciplinary team (MDT) meetings as potentially eligible for the trial. These decisions were shared between the clinical and research team and this assisted in giving the research team confidence in having an open conversation with the potential participant. The research team were given training and assisted by clinicians to provide similar clinical message to all involved participants through their journey in the trial. Clinicians who maintained ongoing involvement with clinical studies and positive relationship with the research staff and accessiblity to the participants generated trust and together helped the recruitment and retention efforts. Studies report that up to 76% of patients expected their physician to alert them about appropriate clinical trials and that physician referral was one of the most useful recruitment strategy [19, 20].

This team approach enhanced the patient’s final understanding of the study as well maintaining equipoise and therefore ultimately increasing the likelihood of participation and randomisation [21]. Similar MDT meetings are in practise in cancer studies and they have found similar results [22].

## Strengths

This is one of the few urogynaecology studies that looked at the complexities in decision-making in women with prolapse prior to embarking on clinical trials. The study has brought out themes that are relevant when planning studies for POP. We were able to identify the factors that facilitate and detract women from participation. We gained insight into the women’s experience and thereby were able to tailor the consenting process. It highlighted the individual differences and the desire for information.

The suggestions voiced by the participants were taken into consideration and helped in framing a definitive trial that was more patient friendly. Two of the women agreed to be part of the patient and public involvement group for the proposed definitive trial.

## Limitations

The views obtained from a small cohort of women who participated in the LOTUS trial. We obtained saturation with a small sample size. However, the research teams did explore and ultimately interviewed 22 women individually and a focused group interview to ensure and verify the emerging themes from individual themes. We do acknowledge there were other women in the trial who participated who may have had unique views and reasons for participation in the trial. In addition it is always challenging to distinguish between various personality types. The study was open label and therefore this could have influenced patient’s experience through the trial process. Some of the themes that emerged were limited to the trial itself and certain elements may not be transferrable outside this setting.

## Conclusion

The benefits of RCTs have been universally appreciated. However, RCTs need to be more sensitive to women’s views and understanding of their trial journey.

From this qualitative study, the researchers found that factors that enhanced participation were maintenance of simplicity in patient leaflets, easy accessibility to research teams and providing clarity in information disseminated, devoid of medical jargon. The barriers to recruitment were uncertainty regarding the investigational product, logistical elements such as physically attending multiple appointments, time constraints and false patient perception of delay in receiving treatment if participating in a clinical trial.

These themes identified in this study will help shape a more efficient and productive definitive study. For successful completion of clinical trials, future vaginal prolapse studies should design their trials keeping the woman’s point of view as paramount importance.
